# Nursing Unit Communication During a US Public Health Emergency: Natural Experiment

**DOI:** 10.2196/11425

**Published:** 2018-12-06

**Authors:** Marge Benham-Hutchins, Kathleen M Carley, Barbara B Brewer, Judith A Effken, Jeffrey Reminga

**Affiliations:** 1 School of Nursing University of Texas at Austin Austin, TX United States; 2 Center for Health Communication Moody College of Communication and Dell Medical School University of Texas at Austin Austin, TX United States; 3 Institute for Software Research School of Computer Science Carnegie Mellon University Pittsburgh, PA United States; 4 College of Nursing The University of Arizona Tucson, AZ United States

**Keywords:** social network analysis, nursing unit communication, Ebola virus disease, public health emergency, natural experiment, nursing

## Abstract

**Background:**

In the second half of 2014, the first case of Ebola virus disease (EVD) was diagnosed in the United States. During this time period, we were collecting data for the Measuring Network Stability and Fit (NetFIT) longitudinal study, which used social network analysis (SNA) to study relationships between nursing staff communication patterns and patient outcomes. One of the data collection sites was a few blocks away from where the initial EVD diagnosis was made. The EVD public health emergency during the NetFIT data collection time period resulted in the occurrence of a natural experiment.

**Objective:**

The objectives of the NetFIT study were to examine the structure of nursing unit decision-making and information-sharing networks, identify a parsimonious set of network metrics that can be used to measure the longitudinal stability of these networks, examine the relationship between the contextual features of a unit and network metrics, and identify relationships between key network measures and nursing-sensitive patient-safety and quality outcomes. This paper reports on unit communication and outcome changes that occurred during the EVD natural disaster time period on the 10 hospital units that had data collected before, during, and after the crisis period.

**Methods:**

For the NetFIT study, data were collected from nursing staff working on 25 patient care units, in three hospitals, and at four data collection points over a 7-month period: Baseline, Month 1, Month 4, and Month 7. Data collection was staggered by hospital and unit. To evaluate the influence of this public health emergency on nursing unit outcomes and communication characteristics, this paper focuses on a subsample of 10 units from two hospitals where data were collected before, during, and after the EVD crisis period. No data were collected from Hospital B during the crisis period. Network data from individual staff were aggregated to the nursing unit level to create 24-hour networks and three unit-level safety outcome measures—fall rate, medication errors, and hospital-acquired pressure ulcers—were collected.

**Results:**

This analysis includes 40 data collection points and 608 staff members who completed questionnaires. Participants (N=608) included registered nurses (431, 70.9%), licensed vocational nurses (3, 0.5%), patient care technicians (133, 21.9%), unit clerks (28, 4.6%), and monitor watchers (13, 2.1%). Changes in SNA metrics associated with communication (ie, average distance, diffusion, and density) were noted in units that had changes in patient safety outcome measures.

**Conclusions:**

Units in the hospital site in the same city as the EVD case exhibited multiple changes in patient outcomes, network communication metrics, and response rates. Future research using SNA to examine the influence of public health emergencies on hospital communication networks and relationships to patient outcomes is warranted.

## Introduction

### Overview

During the second half of 2014, the Ebola virus disease (EVD) epidemic in West Africa became a global public health emergency. On August 5, 2014, the World Health Organization declared EVD an international public health emergency and the US Centers for Disease Control and Prevention (CDC) elevated its Emergency Operations Center to the highest level [1]. On September 30, 2014, the CDC confirmed the first case of EVD in the United States at a Dallas hospital [2]. A few weeks later, two nurses who cared for the patient with EVD were diagnosed with the disease, the first cases of EVD contracted in the United States [1]. During this time period, we were collecting data for the longitudinal study, Measuring Network Stability and Fit (NetFIT), at three acute care hospitals, two in Arizona and one in Texas. One objective of the NetFIT study was to examine the relationship between nursing unit staff communication patterns and patient outcomes. The EVD public health emergency, during the NetFIT data collection time period, resulted in the occurrence of a natural experiment; participants in some hospital units were exposed to situations not controlled by the investigators.

### Background

EVD infection prevention and control presents unique challenges in the health care setting because the virus is present in all body fluids and viral loads increase as the illness progresses [3]. Initial symptoms are similar to common but less serious diseases, so persons under investigation (PUI) for EVD may also pose a risk to health care providers [3]. During the EVD crisis in the United States, press reports often focused on what the nurses who contracted EVD did or did not do that resulted in them becoming infected [4]. Simultaneously, practicing nurses expressed concern about insufficient training and the lack of appropriate personal protective equipment (PPE), which could risk their own personal safety [4]. Shortly after the initial EVD case in the United States, the CDC released new guidelines for PPE and infection control protocols for health care providers caring for PUI or diagnosed with EVD [5]. Nonetheless, many hospitals remained unprepared due to limited training opportunities for staff and PPE availability [6]. Further complicating the situation were quarantine policies, which varied by state, for nurses who had cared for patients with confirmed or suspected EVD. Some policies included up to 21 days in quarantine for nurses who had cared for patients with EVD, resulting in nurses having difficulty balancing their work expectations and personal risk [6].

Safety and quality outcomes and coordination of patient care have been shown to depend on communication among providers in the health care setting [7,8]. Understanding provider communication network characteristics that influence patient outcomes can be an important step toward reducing medical error. Many research methods focus on individual behavior or personality traits, but do not examine interactions between individuals. Social network analysis (SNA) is a research method that supports examination of relationships among individuals, including identification of behavior patterns and the situations in which these patterns arise [9]. In health care, SNA has been widely used to examine provider communication network characteristics. These include patterns of communication on hospital units [10-13] and during patient handoff (ie, transfer of responsibility) between providers during shift change and transfer between units [14,15]. Previous reports from the NetFIT study include the use of SNA to examine the relationship between nursing unit design, staff communication, and patient falls [16] with advice networks, staff information sharing, and patient-safety outcomes [17].

The objectives of the NetFIT study were to examine the structure of nursing unit decision-making and information-sharing networks, identify a parsimonious set of network metrics that can be used to measure the longitudinal stability of these networks, examine the relationship between the contextual features of a unit and network metrics, and identify relationships between key network measures and nursing-sensitive patient-safety and quality outcomes. This paper reports on unit communication and outcome changes that occurred during the EVD natural disaster time period on the 10 hospital units that had data collected before, during, and after the crisis period.

## Methods

### Setting and Sample

For the NetFIT study, data were collected from nursing staff working on 25 patient care units (PCUs), in three hospitals, and at four data collection points over a 7-month period: Baseline (B), Month 1 (M1), Month 4 (M4), and Month 7 (M7). Data collection was staggered by hospital and unit. One unit was dropped from the analysis due to low patient census and staffing. This resulted in 24 units, 96 data collection points, and 1561 licensed and unlicensed nursing staff members who completed questionnaires. To evaluate the influence of this public health emergency on nursing unit outcomes and communication characteristics, this paper focuses on a subsample of 10 units from two hospitals where data were collected before, during, and after the EVD crisis period. The units included in this analysis are Units 5, 6, and 8 from Hospital A and Units 15, 16, 18, 20, 22, 23, and 24 from Hospital C. No data were collected from Hospital B during the crisis period. Table 1 includes the specific data collection time periods and participant response rates for each unit. This analysis includes 40 data collection points and 608 staff members who completed questionnaires. Participants (N=608) included registered nurses (431, 70.9%), licensed vocational nurses (3, 0.5%), patient care technicians (133, 21.9%), unit clerks (28, 4.6%), and monitor watchers (13, 2.1%). Both hospitals are located in urban settings. Hospital A is a not-for-profit institution and Hospital C is a for-profit institution.

### Data Collection

Institutional Review Board (IRB) approval was obtained from the University of Arizona, Texas Woman’s University, the University of Texas at Austin, and the participating hospitals. SNA data collection requires the participants to identify those with whom they have interacted. For that reason, we provided a list of possible contacts—limited to those working on their own units and shifts the day data were collected—for selection by the participants. Our team created a novel data collection system comprised of a secure website, application programming interface, and an Android tablet app. For interested readers, a detailed description of the development and implementation is available [18]. Participants were presented with an IRB-approved study disclosure and consent form at the beginning of the survey. To support participant confidentiality, anonymous IDs were generated by the system and used during the transfer of participant data from the app to the website. Anonymized data could then be downloaded from the website in DyNetML format for network analysis and in standard formats (ie, CSV and XML) for statistical analysis.

For this paper, the starting point for the EVD active crisis period (*during*) was determined by the date when the first EVD case was diagnosed at a Dallas hospital (ie, September 30, 2014). The end point was set as the date when all contacts of this patient completed the 21-day monitoring period (ie, November 7, 2014) [2]. Table 1 provides an overview of response rates, data collection dates, data collection points (ie, B, M1, M4, and M7), and corresponding EVD time period (*before*, *during*, and *after*) for the 10 nursing units included in this analysis.

### Measures

Staff recruitment activities included presentations by research team members during staff meetings and flyers posted on the nursing units. A snack or coupon for a cupcake with a value of US $4.00 was provided to encourage participation. At the end of their shifts, individual attribute (ie, demographic) and SNA data were collected from participating PCU staff working on the designated data collection days. Baseline data were collected on a specific weekday—over a 24-hour period to capture all shifts—and on the same weekday 1, 4, and 7 months later. Network data from individual staff were aggregated to the nursing unit level to create 24-hour networks for network analysis. To create an information-sharing network, participating staff members were asked to identify how frequently they discussed patient care with staff members working on their unit during their just-completed shift. They were also asked how frequently they provided patient care-related information to staff on the next shift or received patient care-related information from staff on the previous shift. To create the decision-making network, staff members were asked how often they sought advice from other staff members, how often other staff sought them out for advice, and to rate their confidence in the advice they received.

**Table 1:** Data collection time periods and individual patient care unit response rates

Hospital, Unit	Baseline (all 2014)		Month 1 (all 2014)			Month 4			Month 7 (all 2015)		
	Date^a^	RR^b^, n (%)		Date	RR, n (%)		Date	RR, n (%)		Date	RR, n (%)
A, 5	6/7-7/30	22/28 (79)		7/28-8/1	25/29 (86)		10/27-10/31, 2014^c^	25/31 (81)		1/26-1/30	30/31 (97)
A, 6	7/7-7/11	24/25 (96)		8/4-8/8	19/21 (91)		11/3-11/7, 2014^c^	17/20 (85)		1/26-1/30	26/28 (93)
A, 8	7/7-7/11	8/9 (89)		8/4-8/8	9/10 (90)		11/3-11/7, 2014^c^	11/11 (100)		1/26-1/30	8/9 (89)
C, 15	6/30-7/4	11/15 (73)		7/28-8/1	12/15 (80)		10/27-10/31, 2014^c^	10/14 (71)		1/26-1/30	9/11 (69)
C, 16	6/30-7/4	15/17 (88)		7/28-8/1	18/19 (95)		10/27-10/31, 2014^c^	13/17 (77)		1/26-1/30	8/19 (42)
C, 18	9/8-9/12	16/21 (76)		10/6-10/10^c^	22/24 (92)		1/5-1/9, 2015	13/20 (65)		3/30-4/3	18/22 (82)
C, 20	9/15-9/19	18/26 (69)		10/13-10/17^c^	16/23 (70)		1/12-1/16, 2015	24/30 (80)		4/6-4/10	22/28 (79)
C, 22	9/22-9/26	12/13 (92)		10/20-10/24^c^	11/15 (73)		1/19-1/23, 2015	7/14 (50)		4/13-4/17	10/15 (67)
C, 23	7/7-7/11	14/20 (70)		8/4-8/8	12/17 (71)		11/3-11/7, 2014^c^	12/14 (86)		2/2-2/6	12/16 (75)
C, 24	9/22-9/26	11/12 (92)		10/20-10/24^c^	7/12 (58)		1/19-1/23, 2015	6/10 (60)		4/13-4/17	10/11 (91)

^a^Data collection dates are reported as month/day, followed by year in the M4 column.

^b^RR: individual patient care unit response rate.

^c^Time period is during active Ebola virus disease period.

**Table 2:** Network metric definitions

Network metric	Definition
Node set size	Total number of nodes (ie, staff members) in the network.
Average distance	The average shortest path between nodes. This statistical measure helps evaluate the efficiency of information transfer.
Clustering coefficient	A higher clustering coefficient indicates a decentralized network and diffusion of information between staff.
Diffusion	Computes the degree to which something could be easily diffused (ie, spread) throughout the network. This is based on the distance between nodes. A large diffusion value means that nodes are close to each other, and a smaller diffusion value means that nodes are farther apart.
Density	Ratio comparing existing links to all possible links in the communication network.
Weighted density	Strength of density connections based on frequency.
Betweenness centralization	Network-level measure that helps identify how dependent the network is on specific providers.
Eigenvector centralization	Network-level measure. High eigenvector centralization indicates that a small group of nodes (ie, staff members) form a group that is fairly densely connected.

Participant characteristics (ie, attributes, also called composition variables) provided contextual information to assist the interpretation of network characteristics [19]. Participants in this study were asked how long they had worked in this hospital and to describe the shift they just worked as *normal*, *better than usual*, or *worse than usual*. In addition, three unit-level safety outcome measures—fall rate, medication errors, and hospital-acquired pressure ulcers (HAPUs)—were collected from the hospital quality-management departments. Fall rate and medication errors were obtained for the month of data collection and defined as the number of falls or medication errors per month divided by patient days, then multiplied by 1000 to create a rate per 1000 patient days. HAPU rates were calculated as the number of HAPUs averaged over the number of patients hospitalized on the unit the day that data were collected.

### Data Analysis

SNA is a distinct research method that supports the study of relationships among actors (ie, nursing unit staff) and analysis of relationship patterns [19]. Sociograms (ie, network graphs) provide snapshot images of the relationship between the nodes (ie, staff) within a bounded social system (ie, nursing unit) [20]. SNA supports node- (ie, individual), dyad-, and network- (ie, unit) level analysis [21]. The unit of analysis for this study was the nursing unit (ie, network level). ORA, a network analysis software program, was used for analyzing relational (ie, network) data [22]. Table 2 provides an overview of the specific SNA metrics used for this study [20,22].

## Results

### Response Rates

On September 30, 2014, when the first patient with EVD in the United States was diagnosed, we had not yet started data collection at Hospital B, but we had completed Baseline and Month 1 data collection at Hospital A. At Hospital C, we had completed Baseline data collection on all 10 units and Month 1 data collection on six of the 10 units. Here we focus on the 10 nursing units with data available before, during, and after the EVD crisis. Table 1 provides the data collection time periods and participant response rates for these units. The units at Hospital A exhibited little change in response rate; in comparison, three units at Hospital C—Units 16, 22, and 24—exhibited major changes in response rate during and after the EVD crisis period. Unit 16 dropped from an 88% (15/17) response rate at Baseline and 95% (18/19) during Month 1 to 77% (13/17) during the active period (M4) and 42% (8/19) during Month 7. Unit 22 dropped from 92% (12/13) at Baseline to 73% (11/15) during the active period (M1) and continued to exhibit poor staff participation during Month 4 (7/14, 50%) and Month 7 (10/15, 67%). By contrast, Unit 24 started with a 92% (11/12) response rate at Baseline, dropped to 58% (7/12) during the EVD crisis period (M1), and 60% (6/10) during Month 4, but recovered for a 90% (10/11) response rate during Month 7.

### Safety Outcome Measures

Table 3 provides an overview of patient-safety outcome measures by time period and nursing unit. Examining outcome measures before, during, and after the EVD crisis periods reveals changes in fall rates, medication error, and HAPU rates. Unit 5, 18, and 20 fall rates increased after the EVD active period (Unit 5, 1.53-8.66; Unit 18, 2.04-4.12; Unit 20, 1.96-3.04) and Unit 24 showed an increase during the active period (1.98-3.98). Medication errors increased during the active period on four nursing units (Unit 5, 3.49-7.66; Unit 6, 3.99-12.62; Unit 15, 1.69-3.59; Unit 16, 1.61-4.12). Medication errors continued to increase for Unit 16 (11.28 at Month 7). Unit 24 did not show an increase during the active period (M1) but this unit’s medication error rate doubled during the Month 4 time period (1.99-4.03) and then the medication error rate dropped during the Month 7 data collection (4.03-2.19). HAPU rates increased on two units during the active time period (Unit 15, 0-4.76 and Unit 18, 0-5.88). The Unit 18 HAPU rate returned to zero during the next data collection period at Month 4, but Unit 16 continued to rise (6.67 at Month 7).

### Network Measures

The NetFIT study was designed to examine the structure of nursing unit decision-making and information-sharing networks. Here we report on the merging of these two networks, as a total interaction network, and the corresponding network metrics (ie, average distance, clustering coefficient, diffusion, density, weighted density, betweenness centralization, and eigenvector centralization). Table 2 provides metric definitions and Table 4 organizes the results by unit and data collection time period [20,22].

Average distance is a measure of information transfer. Two nursing units had increases in average distance during or after the EVD time period. Unit 20 exhibited an increase in average distance during the Month 1 data collection period, which corresponded with the active EVD time period. Unit 16’s Month 1 data collection corresponded with the active EVD time period, but did not show an increase in average distance until the Month 7 data collection period.

The clustering coefficient metric provides information on network characteristics, such as how information spreads between employee groups. A higher metric indicates a decentralized infrastructure and local information diffusion. Standard deviations for each metric, by nursing unit, were calculated using the four data collection time period results (see Table 4). Standard deviation results (0.02-0.11) indicate minimal change during the longitudinal data collection period. The diffusion metric calculates the degree to which something can be spread throughout the network, based on the distance between the nodes (ie, staff). A higher value indicates that nodes are closer to each other [20,22]. The units at Hospital A exhibited consistent diffusion metrics before, during, and after the EVD active period. Hospital C had one unit that was consistent and six units that exhibited changes in diffusion values during and/or after the active EVD period. Units 18, 22, and 24 had decreases in diffusion during and after the EVD time period, while Unit 20 had a decrease in diffusion during the EVD time period. Unit 16 had an increase in diffusion during the EVD time period that dropped afterward, while Unit 23 had an increase in diffusion during the EVD time period but then returned to baseline. Specific metrics are shown in Table 4.

Density is the ratio of all possible links in the network and weighted density indicates the strength of the connections based on how often individual staff indicated they communicated with one another. On nursing Unit 16, the *during* EVD time period coincided with Month 1 of data collection. Density and weighted density decreased during Month 4 and continued to decrease for Month 7 (see Table 4). This indicates a decrease in both the number of connections (ie, links) between staff and the frequency of those connections. The remainder of the units included in this analysis exhibited consistent density and weighted density measures. Betweenness centralization helps identify how dependent the network is on specific providers, while high eigenvector centralization indicates that a small group of staff members are densely connected [20,22]. Both of these measures were consistent for all 10 units before, during, and after the EVD time period.

**Table 3:** Safety outcome measures

Unit	Baseline				Month 1				Month 4			Month 7		
	Fall rate^a^	ME rate^a^	HAPU rate^b^	Fall rate		ME rate	HAPU rate	Fall rate		ME rate	HAPU rate	Fall rate	ME rate	HAPU rate
5	1.74	3.49	0	1.74		3.49	0	*1.53* ^c^		*7.66*	*0*	8.66	3.33	0.67
6	5.43	8.14	1.36	6.66		3.99	0	*4.73*		*12.62*	*0*	2.94	5.87	0
8	0	0	0	0		0	0	*0*		*0*	*0*	0	3.15	0
15	5.08	1.69	0	5.08		1.69	0	*0*		*3.59*	*4.76*	1.79	1.79	6.67
16	0	1.61	0	0		1.61	0	*0*		*4.12*	*0*	0	11.28	0
18	1.17	0	0	*2.04*		*1.02*	*5.88*	4.12		1.03	0	3.38	1.13	0
20	6.47	4.31	2.86	*1.96*		*1.96*	*0*	3.04		0	0	3.31	2.21	7.70
22	5.29	0	0	*1.67*		*0*	*0*	0		0	0	1.70	3.40	0
23	1.77	5.03	0	1.55		0	0	*1.86*		*0*	*0*	1.95	1.95	0
24	1.98	1.98	0	*3.98*		*1.99*	*0*	2.02		4.03	0	0	2.19	0

^a^Fall and medication error (ME) rates were defined as the number of falls or medication errors per month divided by patient days, then multiplied by 1000 to create a rate per 1000 patient days.

^b^Hospital-acquired pressure ulcer (HAPU) rates were calculated as the number of HAPUs averaged over the number of patients hospitalized on the unit the day data were collected.

^c^Outcome measures during the active Ebola virus disease period are in italics.

**Table 4:** Total interaction network measures

Unit and network measure			Baseline		Month 1		Month 4		Month 7		Mean (SD)
**5**											
	Node set size	25		26		*27* ^a^		22		25.00 (2.16)	
	Average distance	0.35		0.29		*0.36*		0.41		0.35 (0.05)	
	Clustering coefficient	0.14		0.11		*0.15*		0.16		0.14 (0.02)	
	Diffusion	0.16		0.24		*0.10*		0.17		0.17 (0.06)	
	Density	0.33		0.29		*0.19*		0.16		0.24 (0.08)	
	Weighted density	0.49		0.42		*0.48*		0.48		0.46 (0.03)	
	Betweenness centralization	3.72		5.06		*4.23*		4.45		4.37 (0.56)	
	Eigenvector centralization	0.74		0.64		*0.81*		0.93		0.78 (0.12)	
**6**											
	Node set size	25		21		*20*		27		23.25 (3.30)	
	Average distance	4.75		5.00		*4.71*		4.13		4.65 (0.37)	
	Clustering coefficient	0.48		0.38		*0.46*		0.51		0.46 (0.06)	
	Diffusion	0.93		0.87		*0.82*		0.94		0.89 (0.06)	
	Density	0.35		0.29		*0.38*		0.41		0.36 (0.05)	
	Weighted density	0.13		0.12		*0.13*		0.15		0.13 (0.01)	
	Betweenness centralization	0.23		0.27		*0.18*		0.30		0.25 (0.05)	
	Eigenvector centralization	0.41		0.30		*0.31*		0.29		0.33 (0.06)	
**8**											
	Node set size	8		10		*11*		9		9.50 (1.29)	
	Average distance	2.58		3.73		*3.23*		3.36		3.23 (0.48)	
	Clustering coefficient	0.62		0.53		*0.69*		0.46		0.57 (0.10)	
	Diffusion	0.96		0.86		*0.97*		0.85		0.91 (0.07)	
	Density	0.64		0.47		*0.67*		0.48		0.57 (0.10)	
	Weighted density	0.33		0.26		*0.30*		0.29		0.29 (0.03)	
	Betweenness centralization	0.10		0.31		*0.13*		0.20		0.18 (0.09)	
	Eigenvector centralization	0.13		0.29		*0.23*		0.22		0.22 (0.07)	
**15**											
	Node set size	15		15		*13*		12		13.75 (1.50)	
	Average distance	3.02		3.76		*2.79*		3.04		3.15 (0.42)	
	Clustering coefficient	0.63		0.51		*0.57*		0.58		0.57 (0.05)	
	Diffusion	0.72		0.78		*0.75*		0.66		0.73 (0.05)	
	Density	0.52		0.44		*0.54*		0.50		0.50 (0.04)	
	Weighted density	0.29		0.26		*0.25*		0.27		0.27 (0.02)	
	Betweenness centralization	0.15		0.16		*0.10*		0.07		0.12 (0.04)	
	Eigenvector centralization	0.19		0.26		*0.17*		0.28		0.23 (0.06)	
**16**											
	Node set size	17		*19*		17		19		18.00 (1.15)	
	Average distance	3.57		*3.60*		4.03		6.51		4.43 (1.40)	
	Clustering coefficient	0.64		*0.61*		0.53		0.41		0.55 (0.10)	
	Diffusion	0.81		*0.93*		0.75		0.34		0.71 (0.25)	
	Density	0.57		*0.54*		0.43		0.20		0.43 (0.17)	
	Weighted density	0.25		*0.22*		0.18		0.06		0.18 (0.08)	
	Betweenness centralization	0.12		*0.26*		0.08		0.12		0.14 (0.08)	
	Eigenvector centralization	0.14		*0.28*		0.36		0.55		0.33 (0.17)	
**18**											
	Node set size	21		*24*		19		21		21.25 (2.06)	
	Average distance	3.12		*3.41*		2.59		2.61		2.93 (0.40)	
	Clustering coefficient	0.53		*0.55*		0.63		0.65		0.59 (0.06)	
	Diffusion	0.84		*0.90*		0.67		0.84		0.81 (0.10)	
	Density	0.43		*0.46*		0.48		0.55		0.48 (0.05)	
	Weighted density	0.24		*0.23*		0.26		0.27		0.25 (0.02)	
	Betweenness centralization	0.20		*0.24*		0.19		0.18		0.20 (0.03)	
	Eigenvector centralization	0.32		*0.34*		0.28		0.29		0.31 (0.03)	
**20**											
	Node set size	26		*23*		30		28		26.75 (2.99)	
	Average distance	3.95		*7.01*		4.57		4.02		4.89 (1.44)	
	Clustering coefficient	0.55		*0.36*		0.52		0.61		0.51 (0.11)	
	Diffusion	0.68		*0.58*		0.82		0.74		0.70 (0.10)	
	Density	0.37		*0.27*		0.39		0.41		0.36 (0.06)	
	Weighted density	0.14		*0.13*		0.15		0.15		0.14 (0.01)	
	Betweenness centralization	0.11		*0.19*		0.16		0.10		0.14 (0.04)	
	Eigenvector centralization	0.29		*0.39*		0.29		0.21		0.30 (0.07)	
**22**											
	Node set size	12		*14*		13		14		13.25 (0.96)	
	Average distance	2.62		*2.55*		2.99		2.57		2.68 (0.21)	
	Clustering coefficient	0.72		*0.62*		0.60		0.59		0.63 (0.06)	
	Diffusion	0.98		*0.77*		0.53		0.70		0.75 (0.19)	
	Density	0.72		*0.57*		0.42		0.46		0.54 (0.13)	
	Weighted density	0.37		*0.28*		0.24		0.25		0.29 (0.06)	
	Betweenness centralization	0.16		*0.09*		0.04		0.24		0.13 (0.09)	
	Eigenvector centralization	0.17		*0.28*		0.25		0.30		0.25 (0.06)	
**23**											
	Node set size	20		17		*14*		16		16.75 (2.50)	
	Average distance	2.75		3.22		*3.38*		3.20		3.14 (0.27)	
	Clustering coefficient	0.57		0.51		*0.67*		0.63		0.59 (0.07)	
	Diffusion	0.69		0.69		*0.84*		0.74		0.74 (0.07)	
	Density	0.41		0.42		*0.63*		0.55		0.50 (0.11)	
	Weighted density	0.18		0.22		*0.30*		0.28		0.25 (0.05)	
	Betweenness centralization	0.40		0.22		*0.27*		0.28		0.29 (0.08)	
	Eigenvector centralization	0.45		0.36		*0.33*		0.29		0.36 (0.07)	
**24**											
	Node set size	12		*11*		10		10		10.75 (0.96)	
	Average distance	3.73		*3.81*		4.31		3.73		3.90 (0.28)	
	Clustering coefficient	0.69		*0.57*		0.45		0.66		0.60 (0.11)	
	Diffusion	0.90		*0.62*		0.58		0.71		0.70 (0.14)	
	Density	0.64		*0.43*		0.35		0.46		0.47 (0.12)	
	Weighted density	0.26		*0.20*		0.15		0.29		0.23 (0.06)	
	Betweenness centralization	0.21		*0.13*		0.31		0.27		0.23 (0.08)	
	Eigenvector centralization	0.28		*0.38*		0.45		0.17		0.32 (0.12)	

^a^Outcome measures during Ebola virus disease active time period are in italics.

## Discussion

### Principal Findings

Individual respondent characteristics, environment, and the timing of data collection have been shown to influence response rates [23]. Researchers studying the relationship between stressors and survey response behavior have proposed that participation in employee surveys is a form of organizational citizenship behavior and is associated with perceptions of organizational support [24,25]. Overload, including perceptions of time availability, has also been associated with completion of surveys [24,25]. Of particular interest when examining reduction in response rate by hospital employees during a public health emergency is the influence of role ambiguity and role conflict [24]. In the same city as Hospital C, two nurses caring for the patient with confirmed EVD contracted the disease despite following CDC and hospital guidelines [6]. Research exploring nurses’ perceptions of caring for patients with EVD or PUI revealed that nurses were fearful of contracting EVD or transmitting it to their families or other patients and lack of training in infection control protocols led to lack of confidence in hospitals [6,26]. Consequently, it is not surprising that units at Hospital C, located in the same city where the first patient with EVD was identified, exhibited major changes in response rate during this time period.

Understanding the negative changes in patient safety outcomes that occurred during the EVD public health emergency also requires examination of corresponding changes to the hospital work environment. The hospital work environment has been shown to influence patient safety outcomes, including rates of falls, medication errors, and HAPUs [27-29]; structural and psychological empowerment of nurses has been shown to support a culture of patient safety [27], which supports positive patient outcomes. Job strain reduces structural and psychological empowerment and is the result of situations in which the nurse has little control [30], such as the EVD outbreak and the resulting psychological demands.

**Figure 1:**
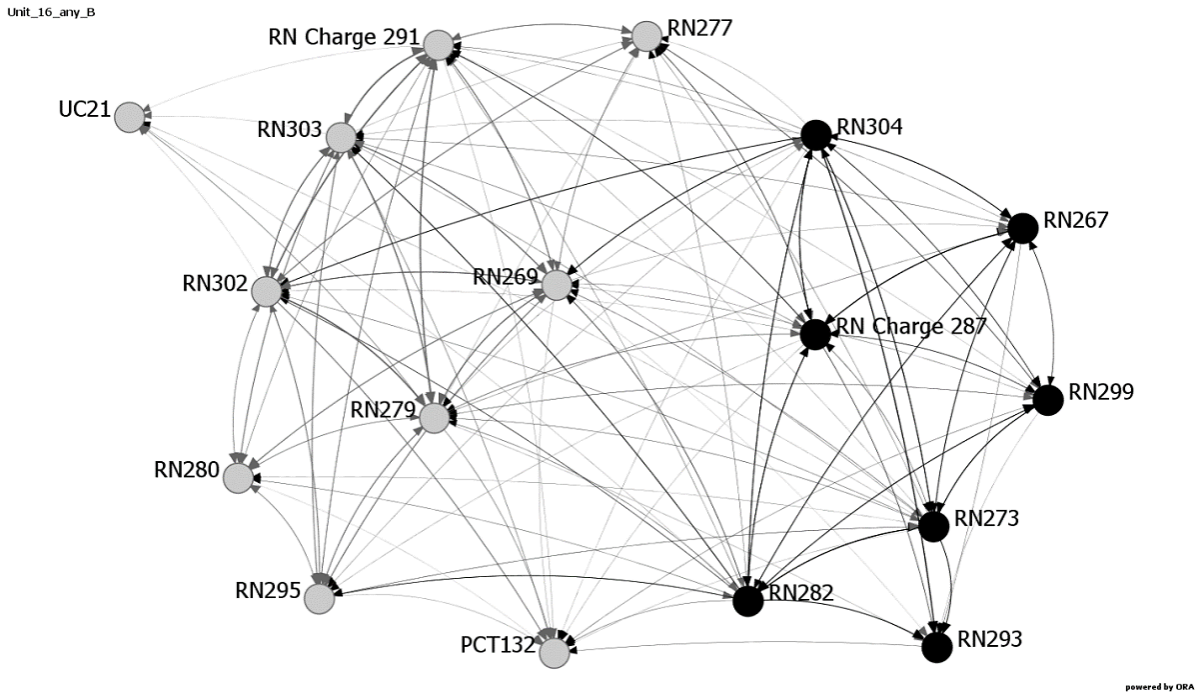
Unit 16, Baseline data collection. The day shift is shown in gray and the night shift is shown in black. The numbers designate the individuals. PCT: patient care technician; RN: registered nurse; RN Charge (head registered nurse); UC: unit clerk.

**Figure 2:**
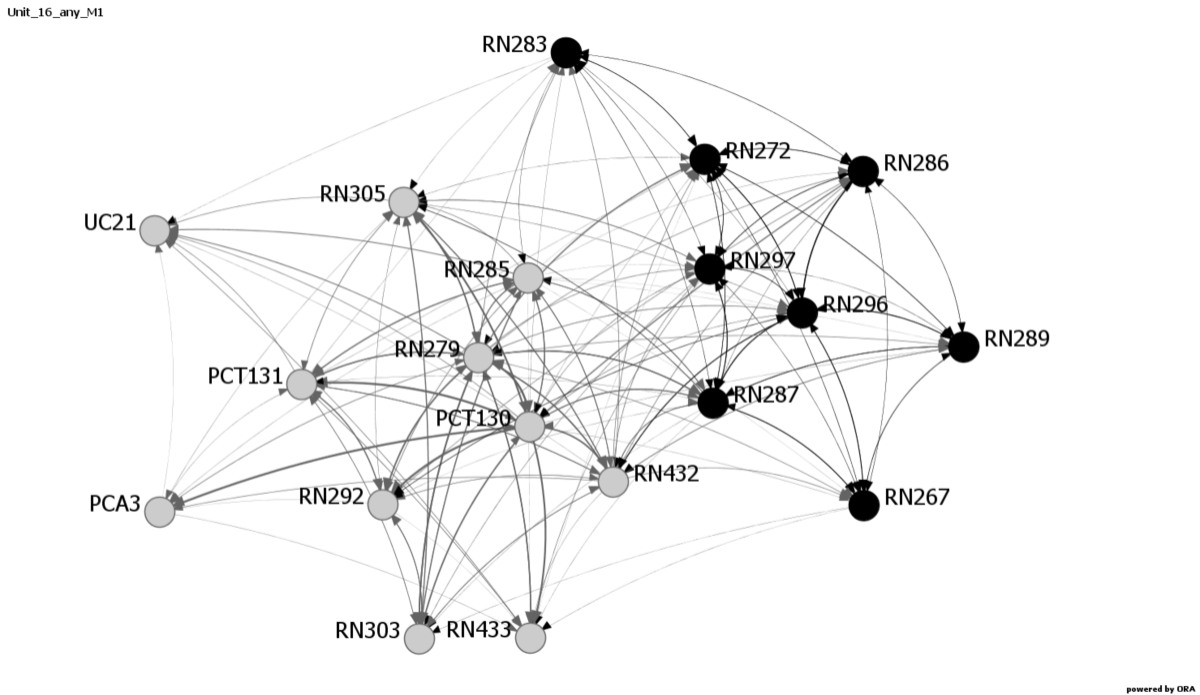
Unit 16, Month 1 data collection. Active Ebola virus disease period. The day shift is shown in gray and the night shift is shown in black. The numbers designate the individuals. PCA: patient care assistant; PCT: patient care technician; RN: registered nurse; UC: unit clerk.

**Figure 3:**
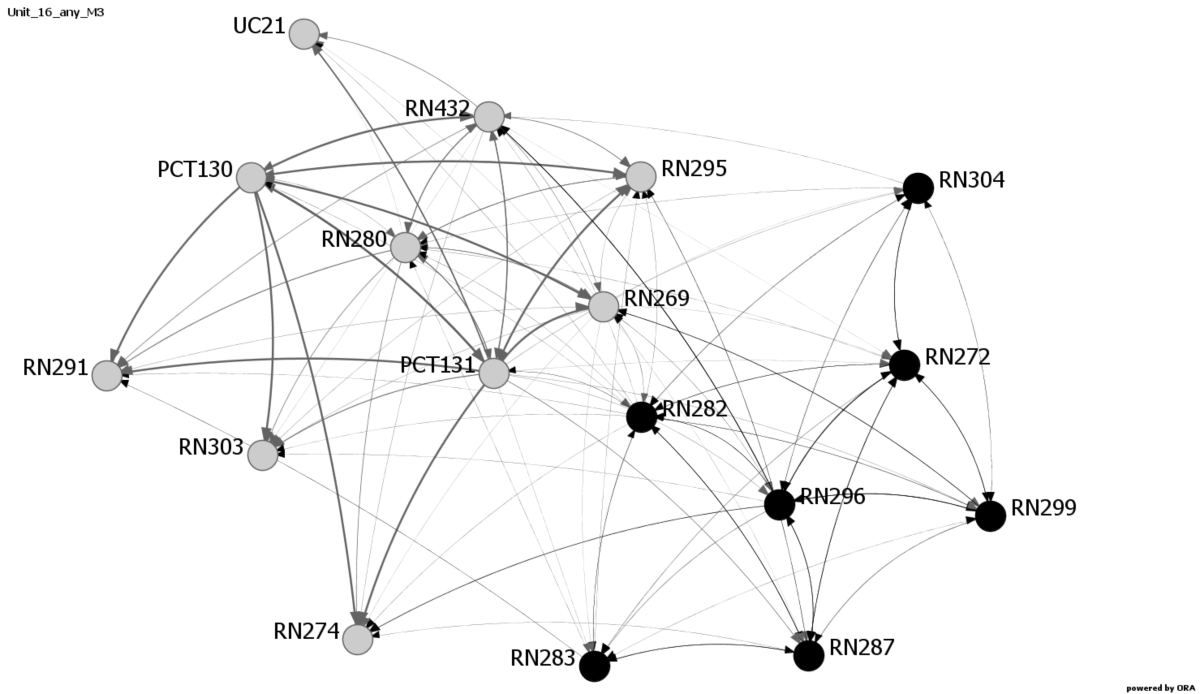
Unit 16, Month 4 data collection. Decrease in diffusion and density. The day shift is shown in gray and the night shift is shown in black. The numbers designate the individuals. PCT: patient care technician; RN: registered nurse; UC: unit clerk.

**Figure 4:**
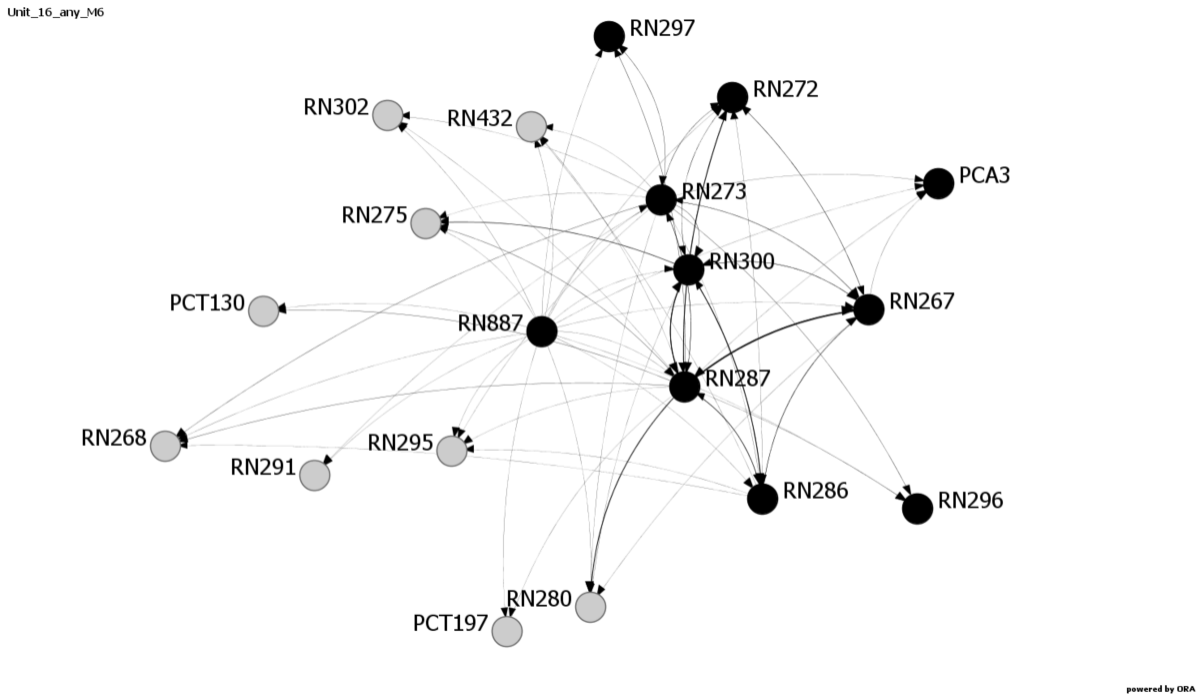
Unit 16, Month 7 data collection. Increase in average distance; decrease in density and diffusion. The day shift is shown in gray and the night shift is shown in black. The numbers designate the individuals. PCA: patient care assistant; PCT: patient care technician; RN: registered nurse.

Communication among providers has been shown to influence patient safety outcomes [7,31]. Research has shown that SNA is a viable way to examine these relationships [10]. Consistent with response rate and patient outcome measure results, the network metrics reveal changes in communication patterns for multiple units at Hospital C. Average distance, diffusion, and density metrics exhibited the most variability during and after the EVD crisis period and reflect changes in communication and network structure on six Hospital C units: 16, 18, 20, 22, 23, and 24. Figures 1-4 provide images of Unit 16 at all four data collection points. Interestingly, Unit 16 is the only unit that had changes in all three metrics—average distance, diffusion, and density—and also showed increases in medication errors during and after the active period, ranging from 1.61 at Baseline and Month 1 to 4.61 at Month 4, followed by 11.28 at Month 7.

### Limitations

Retrospective recognition during data analysis that a natural experiment had occurred may be viewed as a study limitation. Generalizability of the findings is influenced by the specific hospital characteristics and inclusion of only 10 units at two hospitals. We recognize that additional factors, such as unit culture or other local events concurrent with the EVD outbreak, may have influenced the nursing unit network communication patterns and unit outcome measures. However, we had not planned to measure unit culture in the larger study and were unable to measure culture and other possible factors retrospectively.  Although additional research is needed, the viability of using social network analysis to study how external events influence communication and patient outcomes is promising.

### Conclusions

This paper reported on a natural experiment that occurred during data collection for a longitudinal study designed to explore nursing unit communication patterns through the use of social network analysis. The natural experiment occurred when the first case of EVD in the United States was diagnosed in a hospital blocks away from one of our data collection sites. Findings presented in this paper focused on the 10 units that had data collection results available before, during, and after the EVD crisis period. Units in the hospital site in the same city as the EVD case exhibited negative changes in patient outcomes, network communication metrics, and response rates.

## Abbreviations

B: Baseline
CDC: US Centers for Disease Control and Prevention
EVD: Ebola virus disease
HAPU: hospital-acquired pressure ulcer
IRB: Institutional Review Board
M1: Month 1
M4: Month 4
M7: Month 7
ME: medication error
NetFIT: Measuring Network Stability and Fit
PCT: patient care technician
PCU: patient care unit
PPE: personal protective equipment
PUI: persons under investigation
RN: registered nurse
RR: individual patient care unit response rate
SNA: social network analysis
UC: unit clerk
